# Impact of Coronary Collaterals on Infarct Size and Left Ventricular Ejection Fraction After Acute Myocardial Infarction: A Systematic Review and Meta-Analysis

**DOI:** 10.7759/cureus.112963

**Published:** 2026-07-19

**Authors:** Shannon Best, McHenry Mauger, Addison Nguyen, Mohammadali M Shoja, Stephen Ely

**Affiliations:** 1 College of Allopathic Medicine, Nova Southeastern University Dr. Kiran C. Patel College of Allopathic Medicine, Fort Lauderdale, USA; 2 Department of Biostatistics, Nova Southeastern University Dr. Kiran C. Patel College of Allopathic Medicine, Fort Lauderdale, USA; 3 Department of Medical Education, Nova Southeastern University Dr. Kiran C. Patel College of Allopathic Medicine, Fort Lauderdale, USA

**Keywords:** coronary artery disease, coronary collaterals, infarct size, left ventricular ejection fraction, myocardial infarction

## Abstract

Many factors influence outcomes in patients with coronary artery disease (CAD) and acute myocardial infarction (AMI), including patient demographics, comorbidities, cardiac function, clinical presentation, and revascularization therapies. Percutaneous coronary intervention (PCI) is associated with reduced infarct size (IS) and improved left ventricular ejection fraction (LVEF), which have both been shown to reduce all-cause mortality and hospitalization for heart failure following AMI. Coronary collateral circulation may also have a protective effect in CAD and, like PCI, may play a role in limiting IS and improving LVEF after AMI. In this systematic review and meta-analysis, our objective was to investigate the impact of coronary collaterals on IS and LVEF after AMI. A search of Embase, Web of Science, and PubMed from 1980 onwards was conducted in July 2025 using the keywords “coronary collaterals”, “myocardial infarction”, and “infarct size.” A total of six studies (n=820) were included for review, which compared IS and LVEF measured by cardiac magnetic resonance imaging (CMR) between patients with poor and good collaterals after undergoing PCI for AMI. Mean difference (MD) was calculated, and both the common effect and random effects models were conducted to pool effect sizes. IS was consistently larger in patients with poor collaterals. The random effects model gave a statistically significant pooled mean difference (MD) of 5.19 (95% CI = 0.99, 9.40, Z = 3.18, p = 0.024). Studies reported mixed results regarding LVEF, and the random effects model gave us a pooled MD -2.62 (95% CI = -5.36, 0.11, Z = -2.03, p = 0.056), which was not statistically significant. In conclusion, we found that in patients presenting with AMI, greater development of coronary collaterals is associated with a significant reduction in IS. Clinically, this finding is important as a smaller IS is associated with a reduction in long-term mortality and hospitalization for heart failure after AMI treated with primary PCI. We also found a trend toward improved LVEF in patients who experienced AMI with more extensive collaterals, although these results were not significant. Based on these results, the impact of coronary collaterals on IS rather than LVEF may be a more promising area of research for improving patient survival after AMI.

## Introduction and background

Coronary artery disease (CAD) and acute myocardial infarction (AMI) are leading causes of morbidity and mortality in the United States and worldwide [1]. Atherosclerotic plaque development in the coronary arteries may produce flow-limiting stenosis or rupture, resulting in thrombus formation, leading to an AMI [2]. Various demographics and comorbidities are associated with poor clinical outcomes in patients with CAD and AMI, including but not limited to older age, male sex, low socioeconomic status, obesity, diabetes, dyslipidemia, prior myocardial infarction (MI), and history of smoking. Additionally, measures of cardiac function, such as reduced left ventricular ejection fraction (LVEF) and left ventricular hypertrophy, and patient clinical presentation features, such as cardiogenic shock and ST-segment elevation MI (STEMI), are strong predictors of adverse outcomes [3].

Since the 1970s, therapeutic advancements such as the utilization of fibrinolytic agents, percutaneous coronary intervention (PCI), and coronary artery bypass grafting (CABG) have led to a reduction in mortality due to CAD and AMI [2,4]. These therapies restore blood supply and oxygen delivery to ischemic myocardium, thus limiting myocardial damage and preserving cardiac function. For instance, PCI, where available and when performed within 90 minutes of patient arrival at the emergency department, is associated with significant reductions in infarct size (IS) and improvement in LVEF following AMI [5,6]. IS and LVEF are well-established prognostic markers after AMI. Larger IS is strongly associated with mortality, while reduced LVEF is an important predictor of heart failure and major adverse cardiac events [5,7,8].

Collateral circulation may also have a protective effect in CAD and, like PCI, may play a role in limiting IS and improving LVEF after AMI. At birth, the human heart contains precursor anastomotic vessels that connect major coronary arteries or their branches. Under normal conditions, these small vessels carry only minimal blood flow and remain functionally insignificant. However, if obstruction of a coronary artery occurs, as in CAD, these vessels have the potential to expand into functional collaterals [9-13]. Coronary obstruction leads to increased shear stress on the vascular wall, which in turn results in the expression of endothelial chemokines, adhesion molecules, and growth factors. This cascade drives coronary collateral development through arteriogenesis, a process in which pre-existing arterioles transform into mature, functional arteries [9,13-15]. Following acute atherosclerotic plaque rupture and coronary occlusion during AMI, these pre-existing collateral vessels can be rapidly recruited to provide an alternative source of blood supply to the myocardium at risk of ischemia [9,12-15]. Because well-developed coronary collaterals often arise in the setting of longstanding or severe CAD, their presence should be considered both an adaptive protective mechanism and a marker of more advanced underlying disease when interpreting clinical outcomes. 

We hypothesize that an extensive network of pre-existing coronary collaterals is associated with smaller IS and increased LVEF after AMI, thus improving long-term outcomes for patients. Studies exist on the association between coronary collaterals and patient outcomes in CAD and AMI, but they are limited in number and show varied results. Additionally, only a handful focus on IS and LVEF, two clinically important measures of myocardial injury and ventricular function. Therefore, we conducted a systematic review and meta-analysis to investigate the impact of coronary collaterals on IS and LVEF after AMI.

## Review

Methods

This systematic review and meta-analysis followed the framework outlined by the Preferred Reporting Items for Systematic reviews and Meta-Analyses (PRISMA) 2020 guidelines [16]. This review was conducted without prospective protocol registration.

Search Strategy and Eligibility Criteria

A comprehensive search of Embase, Web of Science, and PubMed from 1980 onwards was conducted in July 2025 using the keywords “coronary collaterals”, “myocardial infarction”, and “infarct size” combined with the Boolean operator AND. No controlled vocabulary terms (Medical Subject Headings (MeSH)/Emtree) were used. No publication type restrictions were applied. This search strategy was designed by two researchers (SB, SE). Study selection and screening were performed by a single reviewer (SB) based on predefined eligibility criteria. Studies were included if they involved human patients undergoing PCI for AMI (including STEMI and non-STEMI (NSTEMI)), measured IS and LVEF using cardiac magnetic resonance imaging (CMR) at any time point after AMI, and classified collaterals using the Rentrop classification system [17]. Studies were excluded if they involved non-human subjects or did not meet the predefined inclusion criteria, including studies that did not involve patients undergoing PCI for AMI, did not assess infarct size and/or LVEF using CMR, or did not classify coronary collaterals using the Rentrop classification system.

Data Extraction

Eligible studies were pooled, and data were extracted from articles, tables, and figures by a single reviewer (SB). Data collected included first author, year of publication, location, study design, Rentrop classification, sample size, age, gender, symptom-to-balloon time, and mean and standard deviation of IS and LVEF. If studies reported median and interquartile range (IQR) for IS and LVEF, mean and standard deviation (SD) were estimated using median, quartile 1, quartile 3, and sample size, assuming a normal distribution [18,19].

Risk of Bias Assessment

The included studies were assessed using the Newcastle-Ottawa Scale (NOS), a tool designed to measure quality of evidence and risk of bias in non-randomized observational studies [20].

Statistical Analysis

A meta-analysis was conducted comparing IS and LVEF measured by CMR between patients with poor and good collaterals after AMI. Collaterals were considered poor if they were Rentrop grade 0/1 and good if they were Rentrop grade 2/3. Statistical software used for the meta-analysis was R version 4.2.2. Mean difference (MD) was calculated, and both the common effect and random effects models were conducted to pool effect sizes. The Cochrane Q statistic (I^2^) was used to assess between-study variability, with I^2^>50% considered to be high heterogeneity.

Results

**Figure 1:**
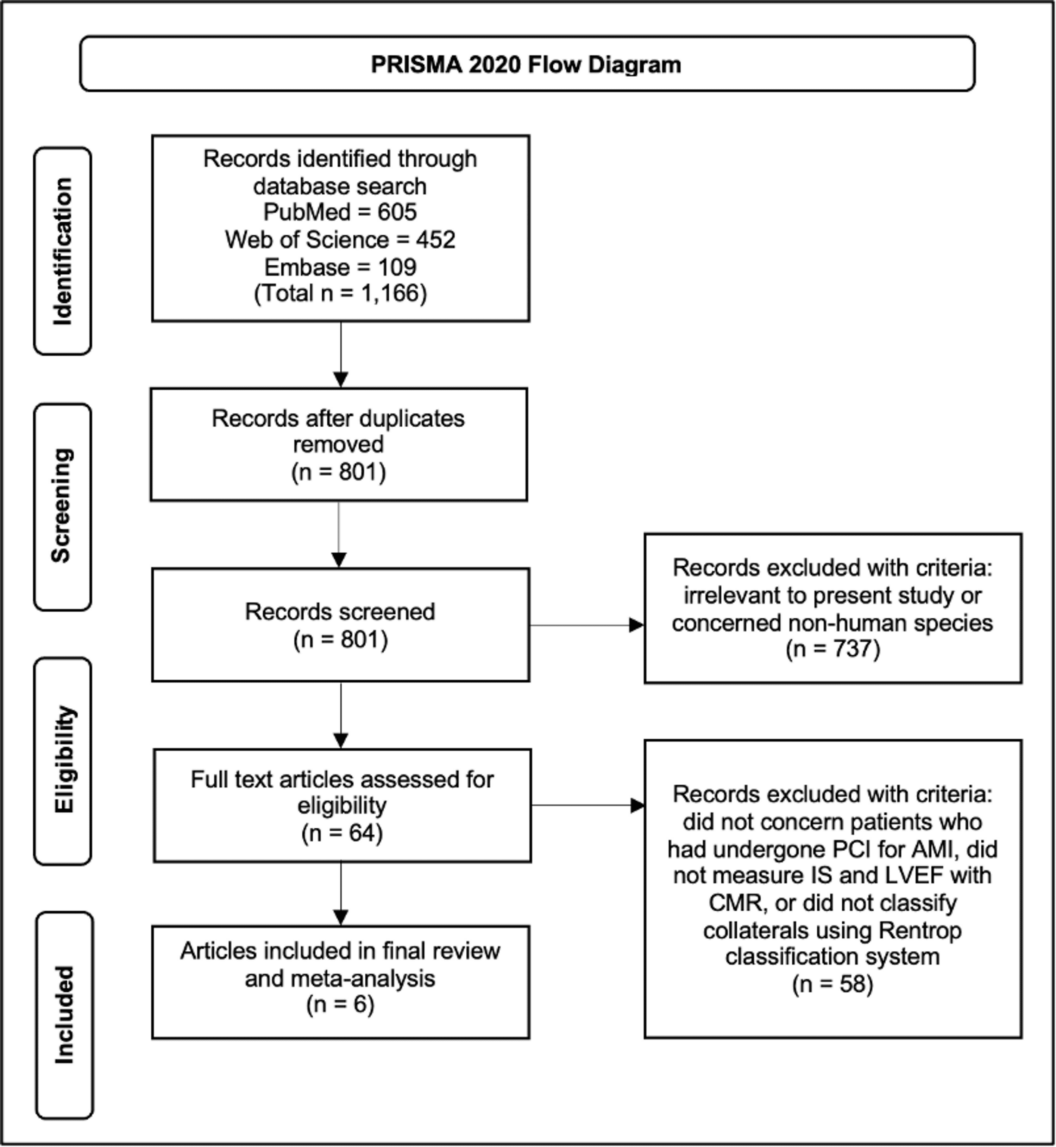
Preferred Reporting Items for Systematic reviews and Meta-Analyses (PRISMA) flowchart

Our search strategy yielded a total of 1,166 articles. As shown in Figure 1, duplicates were removed, as well as 737 articles that were deemed irrelevant to the current study or concerned non-human subjects. Inclusion/exclusion criteria were applied to the remaining 64 articles, of which six met the eligibility criteria and were included in the meta-analysis. In total, 820 patients were included in the meta-analysis.

Table 1 displays the characteristics of the studies that were included. Table 2 displays the results of the NOS quality assessment, which indicated that all six studies were of good quality. The primary areas of potential bias were related to assessment of outcomes and follow-up, while most studies demonstrated adequate selection of study participants and comparability of study groups.

**Table 1:** Summary of data extraction and study characteristics Data are provided as mean±standard deviation or median (interquartile range). LVEF: left ventricular ejection fraction; IS: infarct size

Study	Year	Location	Study Design	Rentrop Grade 0/1						Rentrop Grade 2/3					
				Sample Size (n)	Age	Male (%)	Symptom-to-Balloon Time	IS (%LV)	LVEF (%)	Sample Size (n)	Age	Male (%)	Symptom-to-Balloon Time	IS (%LV)	LVEF (%)
Desch et al. [21]	2009	Germany	Observational cohort study	140	66 (54-74)	76	207 (145–360) minutes	21.8±15.06	45.33±10.94	64	64 (52-73)	74	242 (158–456) minutes	19.73±14.26	45.37±11.07
Freund et al. [22]	2020	Germany	Randomized controlled trial	47	65±16	72	28.2±11.1 hours	34.8±17.2	41.4±8.2	26	66±12	72	28.5±12.4 hours	27.9±11.7	48.7±11.8
Kim et al. [23]	2016	Korea	Observational cohort study	193	58.7±12.3	76.7	188.0 (102.3-356.5) minutes	21.8±10.5	51.57±8.96	54	60.2±10.8	90.7	215 (98.0-640.3) minutes	17.1±10.1	53.23±12.8
Lonberg et al. [24]	2012	Denmark	Observational cohort study	127	62±10	81	190 (125– 287) minutes	11.67±7.5	50.67±9	26	65±7	88	208 (123– 399) minutes	10±6.27	54±8.63
Ortiz Perez et al. [25]	2010	United States	Observational cohort study	65	56±11	83	186 (130–310) minutes	24.4±12.5	39.4±10.4	42	58±10	88	218 (160–511) minutes	19.1±9.2	43.9±9
Pec et al. [26]	2023	Germany	Observational cohort study	28	55±9	86	234 (170–603) minutes	20±12	46±9	8	57±10	63	254 (167–381) minutes	7±4	46±6

**Table 2:** Newcastle-Ottawa scale quality assessment

Study	Selection	Comparability	Outcome	Total	Quality
Desch et al. [21]	****	**	***	9	Good
Freund et al. [22]	****	**	**	8	Good
Kim et al. [23]	****	**	***	9	Good
Lonberg et al. [24]	****	**	***	9	Good
Ortiz Perez et al. [25]	****	**	**	8	Good
Pec et al. [26]	****	*	***	8	Good

**Figure 2:**
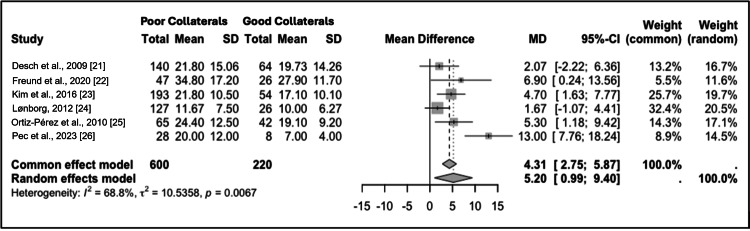
Infarct size forest plot Forest plot of pooled mean differences in infarct size (% LV) between patients with poor (Rentrop 0/1) and good (Rentrop 2/3) coronary collaterals. MD: mean difference References: [21-26]

All six studies reported a larger mean IS in patients with poor collaterals than in patients with good collaterals, with MD ranging from 1.67 to 13.00 (Figure 2). The random effects model gave a statistically significant pooled MD of 5.19 (95% CI = [0.99, 9.40], Z = 3.18, p = 0.024). High heterogeneity was observed (I² = 68.8%, τ² = 10.53, p = 0.006), suggesting between-study variability.

**Figure 3:**
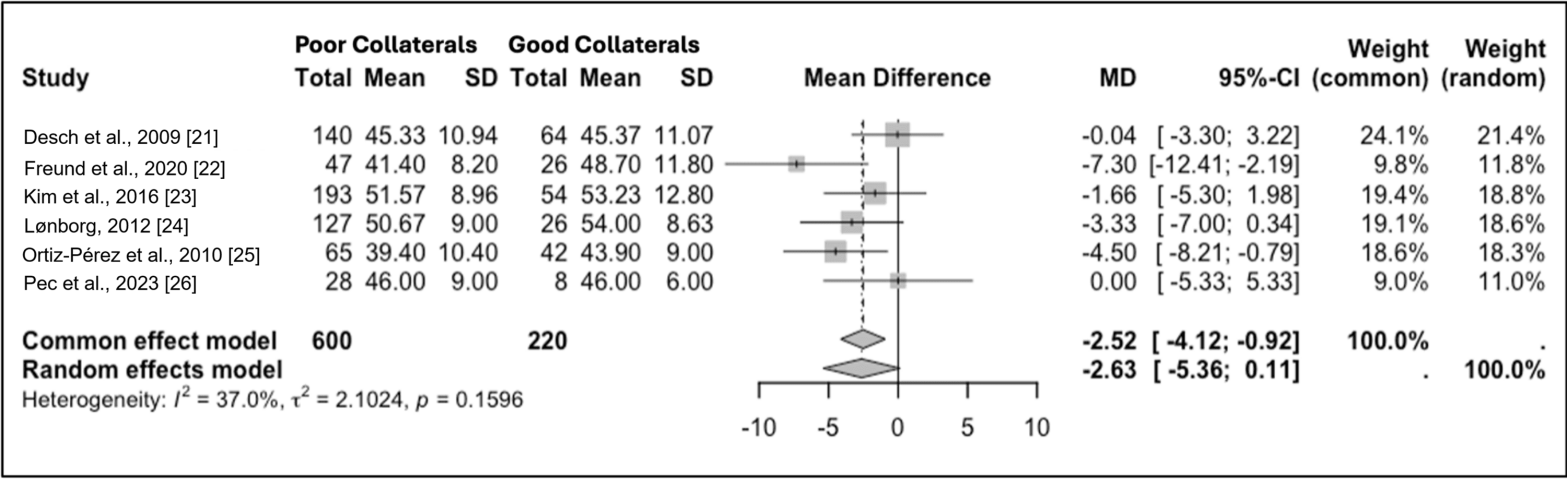
Left ventricular ejection fraction forest plot Forest plot of pooled mean differences in left ventricular ejection fraction (%) between patients with poor (Rentrop 0/1) and good (Rentrop 2/3) coronary collaterals. MD: mean difference References: [21-26]

Two studies [22,25] showed a significantly lower LVEF in the days following AMI in the poor collateral group (MD range: -7.30 to -4.50, p < 0.05), while PEC 2023 found no difference (MD = 0.00, 95% CI -5.33, 5.33, p = 0.999) (Figure 3). The random-effects model gave a pooled MD of -2.62 (95% CI = -5.36, 0.11, Z = -2.03, p = 0.056), which was not statistically significant. The heterogeneity observed was moderate (I² = 37.0%, τ² = 2.10, p = 0.159).

Discussion

The objective of this meta-analysis was to investigate the relationship between coronary collaterals and both IS and LVEF after AMI. The main finding of this study is that in patients presenting with AMI, greater development of coronary collaterals is associated with a significant reduction in IS. This finding suggests that collaterals protect myocardial tissue from ischemia during occlusion of a coronary artery. Collaterals likely do this by providing an alternative source of blood supply and oxygen delivery to at-risk tissue. Clinically, this finding is important as a smaller IS is associated with a reduction in long-term mortality and hospitalization for heart failure after AMI treated with primary PCI [5]. It should be noted, however, that the timing of CMR after AMI may influence measurements of infarct size through changes in myocardial edema, microvascular obstruction, and myocardial salvage [27].

This study also found a trend toward improved LVEF in patients who experienced AMI with more extensive collaterals, although these results were not statistically significant. The variability in these results may be due to CMR measurements that were taken anywhere from one to seven days after treatment. It has been demonstrated that LVEF can take weeks to months to significantly improve in patients who undergo treatment for AMI. LVEF is also influenced by the timing of reperfusion after AMI and the location of infarction, which varied among the patients included in this analysis [28-30].

While preservation of LVEF is associated with fewer major adverse cardiac events following AMI, IS has been shown to be a stronger predictor of long-term mortality [31,32]. Considering this with the results of the current study, the greater impact of coronary collaterals on IS rather than LVEF may be a more promising area of research for improving patient survival after AMI. It has been shown that collaterals develop through arteriogenesis in response to vascular shear stress [9,13-15]. Continued research into the endothelial signaling mediators and mechanisms involved in collateral development may inform future therapeutic options. Inducing coronary collaterals, whether through pharmaceuticals or preconditioning, could be a potential approach to reducing infarct size after AMI and improving long-term patient outcomes. Other approaches to improving outcomes after PCI for AMI may include therapies targeting inflammation, thrombosis, and adverse ventricular remodeling. Anti-platelet and anti-inflammatory therapies, such as colchicine, have been investigated for their potential to reduce recurrent ischemic events and modulate post-PCI inflammatory responses [33]. These complementary approaches emphasize that myocardial protection after AMI is influenced by multiple interacting mechanisms, with coronary collateral development representing one potential component of a broader therapeutic strategy.

Limitations

This review has several limitations that should be considered when interpreting the findings. First, study screening and selection were performed by a single reviewer, which may have introduced potential selection bias despite the use of predefined eligibility criteria. Similarly, data extraction was conducted by a single reviewer, which may have increased the risk of data extraction errors. Second, some included studies reported infarct size and/or LVEF as median and IQR values rather than mean and SD, requiring conversion. Although established methods were used for these conversions, they rely on assumptions regarding the underlying data distribution and may introduce uncertainty into the pooled estimates. Finally, there are many potential confounding factors that could not be accounted for across the included studies. These factors include variability in infarct location, symptom-to-balloon time, timing of CMR measurement, and disease severity. Additionally, the included studies predominantly consisted of STEMI patients, with comparatively fewer NSTEMI patients represented. Differences in infarct characteristics and clinical presentation between STEMI and NSTEMI should be considered when interpreting the results.

## Conclusions

This review investigated the impact of coronary collaterals on IS and LVEF after AMI. Consistent with our hypothesis, patients with greater development of collaterals had significantly smaller IS. These patients also exhibited a trend towards improved LVEF, although these results were not significant. These findings provide evidence that coronary collaterals play a role in limiting myocardial damage after AMI. Further studies are needed to determine whether interventions that promote coronary collateral development are feasible and whether they can translate into reductions in infarct size and improved clinical outcomes after AMI.
